# The Impact of an Interactive Guidance Intervention on Sustained Social Withdrawal in Preterm Infants in Chile: Randomized Controlled Trial

**DOI:** 10.3389/fped.2022.803932

**Published:** 2022-04-01

**Authors:** Jorge Bustamante Loyola, Marcela Pérez Retamal, Andrés Mendiburo-Seguel, Antoine Claude Guedeney, Ricardo Salinas González, Lucia Muñoz, Horacio Cox Melane, José Miguel González Mas, Sandra Simó Teufel, Mónica Morgues Nudman

**Affiliations:** ^1^Clínica Alemana, Universidad del Desarrollo, Santiago, Chile; ^2^Doctoral Programme in Clinical and Health Psychology, University of Valencia, Valencia, Spain; ^3^Association for Infant Mental Health From Pregnancy (ASMI-WAIMH), Valencia, Spain; ^4^Psychology Department, Andres Bello University, Santiago, Chile; ^5^Paris 7, Université Paris Diderot, Paris, France; ^6^Hospital Bichat Claude Bernard, Assistance Publique–Hôpitaux de Paris, Paris, France; ^7^Hospital San José, Santiago, Chile

**Keywords:** social withdrawal, preterm (birth), early detection, emotional distress, interactive guidance, postnatal depression, posttraumatic stress (PTS), social development

## Abstract

**Background:**

Sustained social withdrawal is a key indicator of child emotional distress and a risk factor for psychological development. Preterm infants have a higher probability of developing sustained social withdrawal than infants born full-term during their first year.

**Objective:**

To compare the effect of a behavioral guidance intervention to that of routine pediatric care on sustained social withdrawal behavior in preterm infants.

**Design:**

Multicenter randomized clinical trial.

**Participants:**

Ninety nine moderate and late preterm newborns and their parents were recruited and randomized into two groups, i.e., Intervention (*n* = 49) and Control (*n* = 50). Both groups attended medical check-ups at 2, 6 and 12 months and were assessed with the Alarm Distress Baby Scale. The intervention group received a standardized behavioral intervention if the neonatologist detected sustained social withdrawal. Also, parents filled out the Edinburgh Postnatal Depression Scale, the modified-Perinatal Posttraumatic Stress Disorder Questionnaire, and the Impact of Event Scale-revised.

**Results:**

At baseline, the prevalence of withdrawal was 4.0% (95% CI: 0.03–14.2) for the control group and 22.4% (95% CI: 13.0–35.9) for the intervention group [OR = 0.22, *p* = 0.028 (95% CI =0.06–0.84)]. At 6 months, the prevalence was 10.0% (95% CI: 3.9–21.8) for the control group and 6.1% (95% CI: 2.1–16.5) for the intervention group [OR = 2.09, *p* = 0.318 (95% CI = 0.49–8.88)]. At 12 months, the prevalence was 22.0% (95% CI: 12.8–35.2) for the control group and 4.1% (95% CI: 1.1–13.7) for the intervention group [OR = 6.63, *p* = 0.018 (95% CI = 1.39–31.71)]. Logistic generalized estimating equation models were performed. The pooled crude OR (considering diagnosis at 6 and 12 months) was 3.54 [*p* = 0.022 (95% CI = 1.20–10.44); Cohen's d= 0.70]. In the case of pooled adjusted OR, the model considered diagnosis (0 = Withdrawal, 1 = Normal) as the dependent variable, time of evaluation (1= 6 months, 2 = 12 months) and group (0 = Control, 1 = Experimental) as factors. In this case, the pooled adjusted OR was 3.57 [*p* = 0.022 (95% CI = 1.20–10.65); Cohen's d = 0.70].

**Conclusion:**

Assessment and intervention of sustained social withdrawal in preterm infants via standardized instruments benefits families by reducing its prevalence, and possible associated negative outcomes.

**Clinical Trial Registration:**

ClinicalTrials.gov; https://clinicaltrials.gov/ct2/show/NCT03212547, identifier: NCT03212547.

## Introduction

Infants in the normal range of development have the ability to make contact with the social world around them from birth on (1). During their first 2 months of life, they display skills such as vocalizing, initiating, and holding eye contact, using facial expressions and body movements to engage in and maintain interactions with their caregivers (2) as well as with the caregiving environment around them. Both the capacity to synchronize interactive behaviors and the emotional regulation between infants and their caregivers seems to be critical for optimal psychological development (2, 3).

Preterm newborns often spend their first days, weeks, or even months of life in a neonatal intensive care unit (NICU), where they can be submitted to various perinatal stresses by an environment that demands constant adaptation from the newborns and their parents. It is well known that preterm infant population shows a significantly higher prevalence of psychopathology (4–7); and they also display a higher level of social withdrawal behaviors (8–10) when compared to full-term infants. Also, compared to parents of full-term infants, parents of preterm infants show significantly higher clinical postpartum depression and posttraumatic stress symptoms (PTSS) after the child has been discharged from the NICU (11, 12).

Sustained social withdrawal (SSW) behavior is arguably the first alarm signal of emotional distress displayed by the infant in the first year of life (8–10). Infants can display social withdrawal as an adaptive behavior as a reaction to significant perturbations in the interaction with caregivers (13). SSW, which can be assessed by the Alarm Distress Baby Scale (ADBB) (14), entails a sustained decrease in reactivity to the environment and engagement during interactions (15). When persistent (both repetitive and accumulated), it has shown to be a risk factor for altered emotional development (16) and has been associated with severe psychopathological conditions in infancy (13, 17, 18).

Also, SSW has been linked with medical conditions such as intrauterine growth retardation and preterm birth. In fact, infants born preterm have a higher risk of developing SSW (adjusted odds ratio 1.84, 95% CI 1.04–3.26) when compared to full-term infants (8).

In 2017, of the total population of live newborns in Chile, 8,6% were preterm (19). These infants and their families are assisted by a national network of NICUs and follow-up programs during their first years of life. Considering that preterm birth could imply a risk to psychological development and that interventions become more challenging as problems during infancy are more complex and severe (20), assessing the effectiveness of early SSW detection and intervention in preterm with the ADBB seems to be an interesting and challenging goal.

The ADBB (14) can be used, after certified training, to assess SSW in infants from the age of 2 months and the corrected age of 2 months in the case of preterm infants. It has shown acceptable levels of specificity and sensitivity in several studies (16). Attending medical check-ups with ADBB trained pediatricians who make early detection and intervention, significantly diminished the level of SSW, as shown by the ADBB scores of the full-term infant population (21, 22).

To the best of our knowledge, no studies have been reported regarding the effect of an Interactive Guidance Intervention (IGI) performed by ADBB trained neonatologists on moderate and late preterm infants during their first year of corrected age (23).

Our main objective is to compare the effect of this IGI by assessing the ADBB scores of moderate and late preterm infants vs. routine pediatric care.

## Materials and Methods

### Design and Protocol

This study was designed as a multicenter, randomized, controlled trial (NCT03212547), and its protocol has been previously published (23). The Scientific Ethics Committee of the Research and Clinical essays Unit of the Clínica Alemana de Santiago (Approval Certificate No.201705) approved its protocol. Participants were recruited during their admission to two different neonatal intensive care units by the research team. The researcher presented and explained the informed consent form in case of acceptance. After acceptance, they were randomized and allocated to the intervention or control groups by a study coordinator. One of the neonatology units is part of a private health center located in a district of Santiago, Chile, with an estimated poverty rate of 3.5%. In contrast, the other neonatology unit is a public health center located in a district of Santiago, Chile, with an estimated poverty rate of 20.9% (24).

After discharge, both the intervention and control groups received routine pediatric care during medical check-ups at 2, 6, and 12 months of corrected age. In addition, the intervention group received the IGI, performed by ADBB–trained neonatologists, if they detected SSW (a score of 5 or higher in the ADBB) during these three routine medical check-ups.

Also, medical check-ups of both the intervention and the control groups were video-recorded and two external ADBB–trained evaluators assessed the videos. The study coordinator uploaded the scores to a private online server. Also, on each medical check-up, parents filled out the Edinburgh Postnatal Depression Scale (4), the modified Perinatal Posttraumatic Stress Disorder Questionnaire (5), and the Impact of Event Scale–revised (6). If any parent, considering both control and experimental group, obtained risk scores on depression or posttraumatic stress screenings (4–6) at these medical check-ups, psychiatric evaluation was recommended to them. Before the beginning of this RCT, these assessments were not a standardized part of the routine care protocol included in these medical check-ups.

In case of disagreement in the score categories (normal behavior with a score of 4 or less and sustained social withdrawal with a score of 5 or more) between the two external ADBB-trained evaluators, a third evaluation was made by an external expert to decide.

The families of the infants did not know to which group they were allocated. However, they received feedback on their infant's ADBB score via telephone after the final assessment at the 12-month medical check-up. All infants were offered further assessment and intervention if they had a score of 5 or higher ADBB at 12-month corrected age.

### Description of the Intervention

As explained in the study protocol published by Bustamante et al. (23), before the beginning of the recruitment, the neonatologists of the research team were trained by an expert certified trainer. The training consisted of 30 hours and 12 modules, two of which were theoretical modules that covered early interactions and emotional development, emotional deprivation and its consequences, and social withdrawal behaviors as an early alarm signal of emotional distress. Subsequently, 10 video training modules were presented to learn the ADBB coding system. After completing the 12 modules, every trainee passed an exam to be certified.

One fundamental element of the training was that the professionals learned not only to detect the SSW by scoring these behaviors using the ADBB but also to intervene at a behavioral level when they detected SSW during the medical check-ups. The ADBB scale and the behavioral intervention (IGI) have two different but complementary objectives. The ADBB focuses on detecting (and scoring) the inhibition of some early interaction skills of the infant, and the IGI consists of facilitating a more synchronized interaction between the withdrawal infant and their parents by detecting and highlighting every emotional and behavioral resource displayed from both, infant and caregiver.

The intervention included four key elements:

1. IGI (behavioral intervention): during the medical check-ups scheduled at 2, 6 and 12 months of corrected age, the ADBB-trained neonatologists, who provided care for the intervention group, assessed the infants using the ADBB scale. If they detected SSW (with a score of 5 or higher) they carried out a behavioral intervention (the IGI), which consisted first of synchronizing their interaction rhythm and emotional state with the interaction rhythm and emotional state of the withdrawal preterm infants. In this process, they tried not to avoid or overstimulate the withdrawn infant, kept an attentive wait, and soothed the infant. While the withdrawn infant responded to the intervention and started to display interaction skills (for example, engaging through eye contact or vocalizing), the trained neonatologist explained to the parents how their infants seek and regulate interactions in terms of communication or contact. All the interventions were done during the 20–30 min of these medical check-ups. Considering that there were only three medical check-ups scheduled for this study, each withdrawal infant was intervened a maximum of three times.
2. Written guidelines: at the end of the behavioral intervention, the ADBB trained professionals explained the “ADBB early interaction guidelines” to the parents to enhance the intervention between medical check-ups.
3. Intervention group meetings: the group of neonatologists that provided care for the experimental group attended monthly meetings, guided by an expert ADBB trainer, to watch videos of medical check-ups of infants with SSW. These videos, selected in advance by the ADBB trainer, met one or more of the following criteria: high difficult to code, with scores near the screening cut point (scores from 3 to 7, considering that 5 is the cut point of the ADBB scale); high difficult to intervene, with infants resistant to the IGI; and successful IGI, with withdrawal infants that responded to the IGI. On these meetings, the research team reviewed the correct coding system of the ADBB scale and observed the behavioral interventions made on these videos, with the objectives of homogenizing the efficient behavioral interventions and sharing the difficulties related to the IGI.

### Participants, Randomization, and Allocation

#### Inclusion and Exclusion Criteria

Eligible preterm infants were those that were born between 32 weeks-0 days and 36 weeks-6 days of gestational age from single or twin pregnancy (monochorionic or dichorionic) and hospitalized within the first 48 h after birth.

Given that Spanish-speaking neonatologists carried out the behavioral intervention, it was required that the parents speak Spanish.

Infants with major congenital malformations, confirmed neurological disease that impaired development, suspected or confirmed genetic disorders, perinatal asphyxia occurring at birth (Apgar score <3 at 1 min or Apgar score <5 at 5 min, or cord pH <7.0 at birth), or with mothers who had a history of exposure to illicit mind-altering substances during pregnancy, were excluded.

#### Sample Size, Randomization, and Allocation

The necessary sample size was estimated using G^*^Power 3 (version 3.1.9.6) (25). Considering the previous results reported by Bonifacino et al. (21), with an α = 0.05, statistical power of 0.95, and a 1:1 allocation ratio, the estimated necessary sample size was 46 participants, 23 per group.

Ninety-nine infants were recruited during their admission to the neonatal intensive care units by research team members and enrolled and randomized by a study coordinator to either the intervention [49] or the control group [50]. The infants were randomized in a 1:1 allocation, stratified by center in four blocks. They were also stratified into two groups, single or twin pregnancy, to control the intervention effect from other covariables (such as mothers of twins learning). In the case of one of the health centres (Hospital San Jose), the sample was also stratified into two groups depending on whether the infants were included in the Kangaroo Care program, designed to promote mother-infant bonding and could also potentially act as a confounding variable.

**Figure 1:**
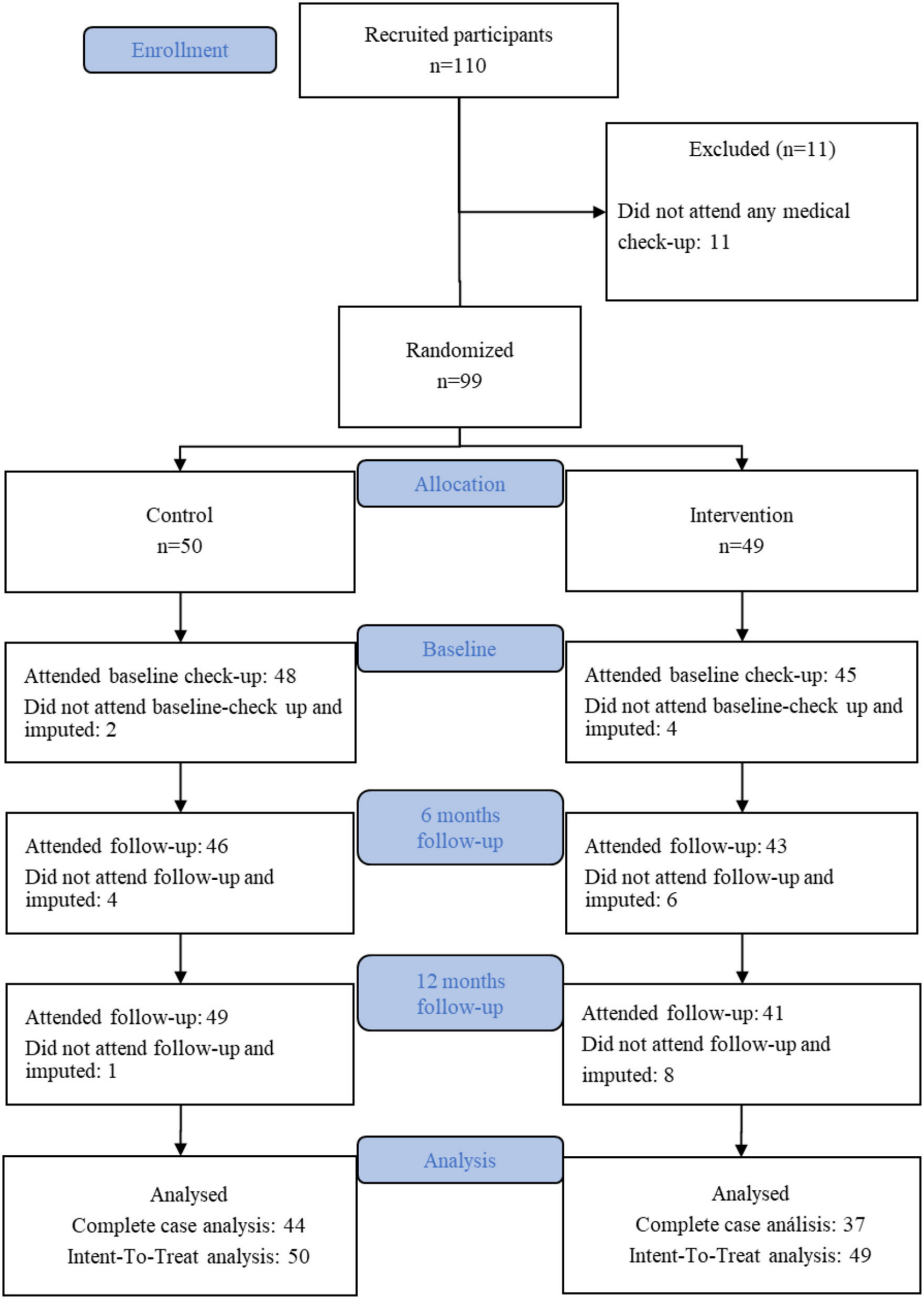
CONSORT diagram of the study.

Of the randomized infants, 11 were not assessed at any moment of the study. We were unable to contact the parents as some changed their contact numbers, did not reply to emails, while others confirmed the assistance but missed up to three appointments. Figure 1 shows the CONSORT flow diagram of the study.

#### Instruments and Measurements

The ABDD (14) assesses sustained social withdrawal behavior in infants from 2 to 24 months of corrected age during routine physical examinations. It consists of eight items (lack of facial expression, eye contact, general movement, self-stimulation gestures, vocalization, liveliness in response to any stimulation, ability to establish and maintain a relationship, and ability to attract and catch the attention of others), each scored from 0 (normal behavior) to 4 (massively abnormal behavior). A total score of 5 or more indicates SSW behavior. The assessment can be done by a trained professional during routine pediatric check-ups or by assessment of an 8–12-min video of recorded infant behavior during a pediatric check-up.

Edinburgh Postnatal Depression Scale (EPDS) (26) assesses the probability of postnatal depression in women. It consists of 10 questions with four possible answers for each. Each answer is given a score of 0, 1, 2, or 3 according to the severity of the symptom. The maximum score is 30. A total score of 12 or higher suggests postnatal depression disorder. The Edinburgh Postnatal Depression Scale can be administered 2 months after delivery and onward.

Impact of Event Scale-Revised (IES-R) (27) assesses symptoms associated with posttraumatic stress disorder. It comprises 22 items and three subscales: intrusion, avoidance, and hyperactivation. It employs a 5-point Likert scale from 0 (not at all) to 4 (extremely) to assess the intensity of the symptoms. The IES-R can be applied 6 weeks after a stressful or traumatic event. Scores higher than 24 indicate significant clinical relevance.

The Modified Perinatal Posttraumatic Stress Disorder Questionnaire (PPQ) (28) assesses parents' posttraumatic stress symptoms, including intrusiveness or re-experiencing, avoidance behaviors, and hyperarousal or numbing of responsiveness. It consists of 14 items, measured using a 5-point Likert scale from 0 to 4. Parents are instructed to provide responses that reflect their experience during the 4th and 18th months after delivery. The total score can range from 0 to 56. The clinical range for a high-risk parent is set at 19 or higher.

At enrolment, an ad hoc demographic survey of parents regarding socioeconomic, pregnancy, and postpartum variables was distributed and results were recorded.

We also registered variables related to each infant: gender, gestational age (weeks), weight (grams), and hospitalization days (number of days).

#### ADBB Interrater Agreement and Reliability

We analyzed the interrater agreement of the ADBB for each stage (baseline and follow-ups) in three different ways. The first considered the total raw scores given by both ratters, using the intraclass correlation coefficient (ICC) under the random-effect model. The second considered the three possible diagnostic levels (normal, moderate withdrawal, severe withdrawal), using weighted kappa (κw) with linear weights. Finally, the third considered the two possible diagnoses used in the study (normal or withdrawal), using Cohen's *Kappa* coefficient. In all cases, the inter-rater agreement was satisfactory (see Table 1).

**Table 1:** Inter-rater agreement for raw scores, two-level and three-level diagnosis at 2, 6, and 12 months.

	**Raw scores (ICC)**	**Three-level diagnosis (κw)**	**Two-level diagnosis (κ)**
2 months	0.86	0.84	0.92
6 months	0.76	0.93	0.92
12 months	0.88	0.89	0.87

The analysis per item of the ADBB scale shows that when it is considered on a global scale, its internal consistency (Cronbach's alpha) is satisfactory at baseline (α = 0.92), 6 months (α = 0.84), and 12 months (α = 0.89).

### Statistical Analyses

We structured the results in four parts.

The first part focused on determining the efficacy of the randomization process and examining the possibility that sample loss could be an effect of the treatment. To evaluate the effectiveness of the randomization process, we compared the intervention and control groups in relation to different possible confounding variables using the chi-square test of independence and Student's *t-*test for independent samples. We then compared participants who were randomized and participated in the study (*n* = 99) with those who had been enrolled but were excluded (*n* = 11), and between those who had some imputed measurement (*n* = 18) and those who had no imputed data (*n* = 81). Finally, we examined the possible influence of the previous diagnosis on missing assessments at 6 and 12 months.

The second part of the analysis sought to examine differences between infants according to their diagnosis (SSW or not) at each stage to determine the possible influence of other variables on the diagnosis. For this, we used the chi-square test of independence and Student's *t-*tests for independent samples.

The third part of the analysis focused on determining the effectiveness of treatment by comparing the intervention group and the control. For this, we analyzed the data using Intent-To-Treat with Last Observation Carried Forward (LOCF) and within-group simple mean imputation (for participants with missing values at baseline but with complete follow-ups). In this comparison, we used odds ratios, and following the recommendation by Twisk, et al. (29) logistic generalized estimating equation (GEE) models to assess the longitudinal effects of treatment in diagnosis.

Finally, given the importance that the literature assigns to caregivers, in the fourth part of the results, we correlated different parents-related variables (postnatal depression, posttraumatic stress symptoms) with the ADBB scores of the infants using bivariate Pearson correlations.

## Results

### Effectiveness of the Randomization Process, Comparison Between Excluded and Randomized, and Comparison Between Participants With Imputed and Non-imputed Data

Before carrying out the core analyses of the study, we examined the effectiveness of allocation and possible differences between the imputed and non-imputed cases. As it can be observed in Table 2, there were no differences between both groups when considered imputed and non-imputed cases.

**Table 2:** Comparisons between conditions and imputed/non-imputed.

	**Control (*N* = 50)**	**Intervention (*N* = 49)**	***p*-value *SMD***	**Imputed (*N* = 18)**	**Non-imputed (*N* = 81)**	***p*-value**	**Total**
Gender			0.484^a^			0.813^a^	
Male	28 (56.0%)	24 (49.0%)	0.156	9 (50.0%)	43 (53.1%)		52 (52.5%)
Female	22 (44.0%)	25 (51.0%)		9 (50.0%)	38 (46.9%)		47 (47.5%)
Gestational age			0.076^b^			0.342^b^	
M (SD)	33.5 (1.1)	33.9 (1.1)	0.361	33.94 (1.3)	33.67 (1.1)		33.7 (1.1)
Range	32–36	32–37		32–37	32–36		32–37
Weight (g)			0.125^b^ 0.311			0.398^b^	
M (SD)	2028.2 (396.7)	2144.9 (351.3)		2154.3 (244.8)	2070.8 (400.8)		2086.0 (377.6)
Range	1285–3313	1500–2960		1870–2680	1285–3313		1285–3313
Hospitalization (days)			0.476^b^			0.098^b^	
M (SD)	19.4 (9.1)	20.9 (11.9)	0.144	23.89 (10.8)	19.35 (10.4)		20.2 (10.5)
Range	5–44	6–60		9–43	5–60		5–60

a *χ^2^*.

b *Student's t-test for independent samples*.

*SMD = Standardized Mean Difference (Cohen's d)*.

First, considering infants who were part of the final analysis, we started by examining the association of allocation with possible pre-considered confounding variables. No associations were found between allocation and center (χ^2^_(1)_ = 0.50, *p* = 0.480, *Cohen's d* = 0.157), single or twin pregnancy (χ^2^_(1)_ = 0.24, *p* = 0.622, *Cohen's d* = 0.114), and participation in the *Kangaroo care* program (χ^2^_(1)_ = 1.66, *p* = 0.198, *Cohen's d* = −0.382). We also compared the samples from the control and intervention groups that were part of the final analysis regarding gender, gestational age, weight, and total days hospitalized. As it can be observed in Table 3, there were no differences between both groups and effect sizes were small or very small. These results confirm a successful randomization process and suggest adequate control of confounding variables.

**Table 3:** Comparison of infants with and without SSW depending on stage and allocation.

**Stage**	**Allocation**	**Diagnosis**	**Male**	**Female**	**Gestational age**	**Weight**	**Hospitalization days**
Baseline	Control	*W*	0	2	34.00 (0.00)	1,995.00 (148.49)	22.00 (8.49)
		*N*	27	19	33.48 (1.17)	2,025.72 (412.36)	19.52 (9.29)
		*p*	0.101		0.535	0.918	0.713
	Intervention	*W*	6	5	33.73 (1.01)	2,153.82 (404.25)	18.82 (9.78)
		*N*	16	18	33.97 (0.97)	2,161.47 (354.29)	20.56 (12.32)
		*p*	0.666		0.477	0.952	0.672
6 months	Control	*W*	2	2	34.25 (0.96)	1,996.25 (526.38)	17.00 (7.96)
		*N*	24	17	33.39 (1.18)	2,028.73 (411.36)	19.54 (9.42)
		*p*	0.741		0.176	0.883	0.606
	Intervention	*W*	2	1	34.00 (1.00)	2,151.67 (372.20)	14.33 (5.13)
		*N*	17	19	34.00 (0.89)	2,159.00 (387.71)	19.67 (11.65)
		*p*	0.517		1	0.975	0.441
12 months	Control	*W*	4	6	33.50 (1.08)	1,957.90 (338.51)	20.00 (9.04)
		*N*	23	14	33.46 (1.17)	2,030.65 (421.93)	19.43 (9.46)
		*p*	0.209		0.922	0.618	0.866
	Intervention	*W*	1	1	33.50 (0.71)	2,128.00 (605.28)	22.00 (21.21)
		*N*	16	19	33.97 (0.89)	2,136.37 (375.11)	19.34 (11.35)
		*p*	0.906		0.469	0.976	0.758

*W, Withdrawal; N, Normal. P-values for Chi squared test (Gender) and t-test*.

Second, we compared participants who were part of the final analyses (*n* = 99) with those who were not part of it for not having baseline nor follow-up assessments (*n* = 11). No differences were found regarding gender (χ^2^_(1)_ = 1.03, *p* = 0.309), gestational age ($Welch^{\prime }st_{\left(12.77\right)}$ = 0.25, *p* = 0.810), weight ($Welch^{\prime }st_{\left(11.36\right)}$ = 1.26, *p* = 0.232), or hospitalization days ($Welch^{\prime }st_{\left(14.12\right)}$ = 0.72, *p* = 0.485).

We also examined the possible influence of the previous diagnosis on missing assessments at 6 and 12 months, observing no differences between infants with complete and missing assessments at 6 months [χ^2^(1) = 0.07, *p* = 0.794] and at 12 months [χ^2^(1) = 0.28, *p* = 0.595]. This indicates that there is no association between previous diagnosis and missing assessments. In order to determine the presence of systematic variation causing attrition during follow-ups, we compared imputed and non-imputed cases regarding the same variables. As in the case of the allocation, no differences were found between complete and incomplete (imputed) cases (Table 3). Imputation was also not associated with allocation, χ^2^_(1)_ = 2.60, *p* = 0.107.

Finally, considering the scores in the three measurements, we used Little's test (Little, 1988) to determine whether the missing data were completely at random. The result indicated that it was, $\chi_{\left(28\right)}^{2}$ = 34.44, *p* = 0.187.

### Differences Between Infants With and Without Sustained Social Withdrawal

In order to have an overview of the differences between infants with and without SSW, we compared both groups at each stage in relation to their gender, gestational age, weight, and hospitalization days. In this way, it was possible to observe that none of these variables was associated with the diagnosis at each stage, as shown in Table 3.

### Description of Sustained Social Withdrawal and Comparison Between Intervention and Control Groups

**Figure 2:**
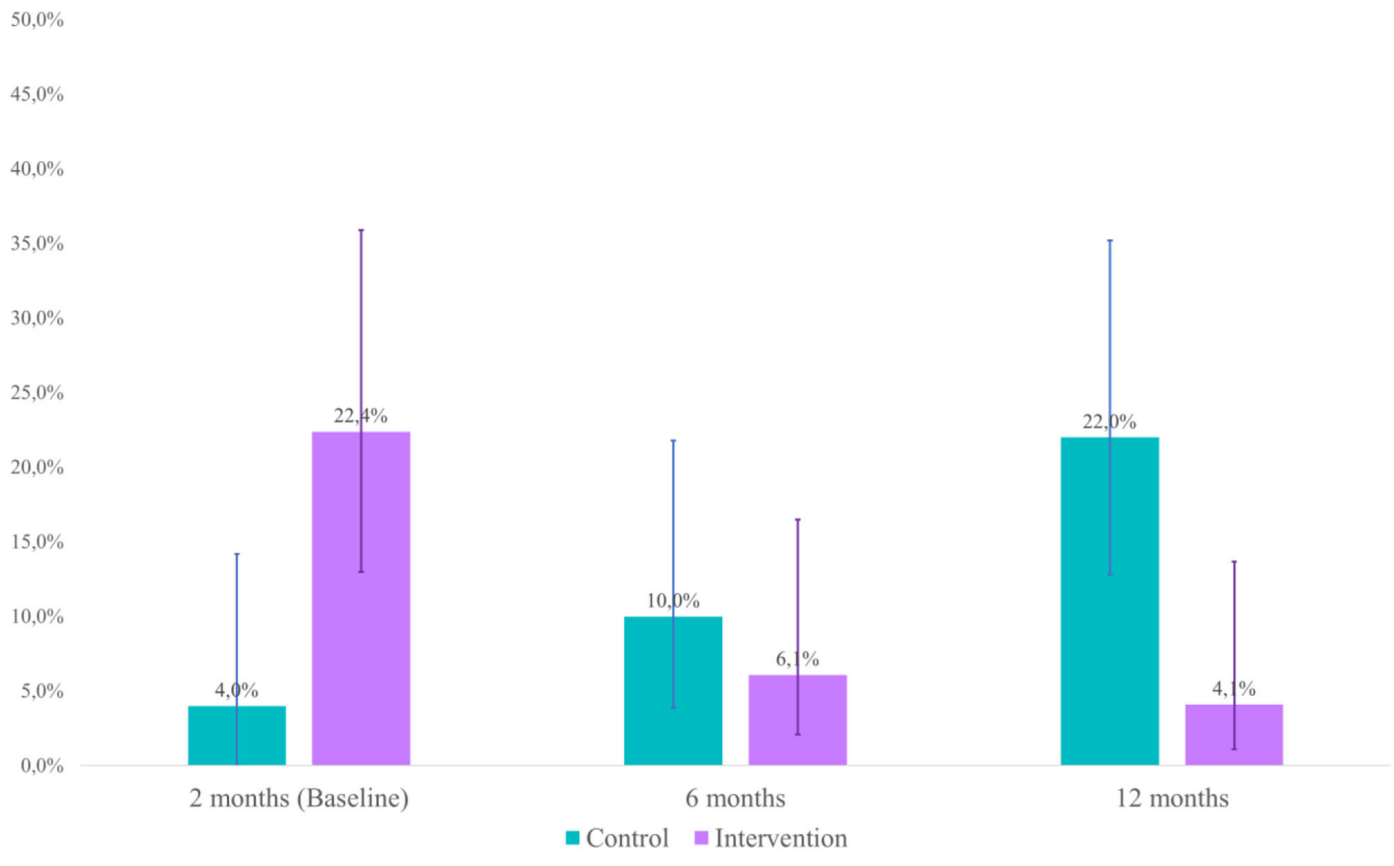
Prevalence of withdrawal (95% CI).

At baseline, the prevalence of withdrawal was 4.0% (95% CI: 0.03–14.2) for the control group and 22.4% (95% CI: 13.0–35.9) for the intervention group [OR = 0.22, *p* = *0*.028 (95% CI =0.06–0.84)]. At 6 months, the prevalence was 10.0% (95% CI: 3.9–21.8) for the control group and 6.1% (95% CI: 2.1–16.5) for the intervention group [OR = 2.09, *p* = 0.318 [95% CI = 0.49–8.88)]. At 12 months, the prevalence was 22.0% (95% CI: 12.8–35.2) for the control group and 4.1% (95% CI: 1.1–13.7) for the intervention group [OR = 6.63, *p* = *0*.018 [95% CI = 1.39–31.71)]. Differences between groups at baseline, 6, and 12 months can be observed in Figure 2.

The pooled crude OR (considering diagnosis at 6 and 12 months) was 3.54 [*p* = 0.022 (95% CI = 1.20–10.44); *Cohen's d*= 0.70]. In the case of pooled adjusted OR, the model considered diagnosis (0 = Withdrawal, 1 = Normal) as the dependent variable, time of evaluation (1 = 6 months, 2 = 12 months) and group (0 = Control, 1 = Experimental) as factors. In this case, the pooled adjusted OR was 3.57 [*p* = 0.022 (95% CI = 1.20–10.65); *Cohen's d*= 0.70].

### Relations Between Parents' Postnatal Depression, Posttraumatic Stress Symptoms, and Infants' ADBB Scores

Table 4 shows the correlations between the ADBB scores and the scores of both parents on the EPDS, IES-R, and PPQ scales. In the case of the mothers, there is a positive correlation between the ADBB at 6 months with the EIE-R assessments at 2 months, while in the case of the fathers there is a positive correlation with the PPQ scale at the same time. Finally, there is a positive correlation at 12 months between the fathers' IES-R scores and the ADBB.

**Table 4:** Correlations between ADBB, EPDS, IES-R, and PPQ scores at 2, 6, and 12 months.

			**EPDS**				**IES-R**				**PPQ**				
			**Mother**			**Mother**			**Father**			**Mother**		**Father**	
			**2m**	**6m**	**12m**	**2m**	**6m**	**12m**	**2m**	**6m**	**12m**	**6m**	**12m**	**6m**	**12m**
EPDS	Mother	6m	0.70^***^												
		12m	0.55^***^	0.72^***^											
IES-R	Mother	2m	0.79^***^	0.69^***^	0.62^***^										
		6m	0.56^***^	0.74^***^	0.64^***^	0.66^***^									
		12m	0.47^***^	0.67^***^	0.83^***^	0.64^***^	0.71^***^								
	Father	2m	0.60^***^	0.58^***^	0.42^***^	0.64^***^	0.46^***^	0.44^***^							
		6m	0.10	0.48^***^	0.39^**^	0.27^*^	0.37^**^	0.59^***^	0.73^***^						
		12m	0.02	0.32^**^	0.36^**^	0.18	0.28^*^	0.48^***^	0.64^***^	0.79^***^					
PPQ	Mother	6m	0.55^***^	0.67^***^	0.62^***^	0.57^***^	0.88^***^	0.69^***^	0.30^*^	0.33^**^	0.26^*^				
		12m	0.11	0.46^***^	0.37^***^	0.31^*^	0.35^**^	0.47^***^	0.70^***^	0.81^***^	0.82^***^	0.34^**^			
	Father	6m	0.10	0.51^***^	0.31^*^	0.21	0.46^***^	0.48^***^	0.53^***^	0.78^***^	0.69^***^	0.46^***^	0.81^***^		
		12m	0.59^***^	0.58^***^	0.80^***^	0.65^***^	0.54^***^	0.82^***^	0.48^***^	0.42^***^	0.40^***^	0.58^***^	0.41^***^	0.33^**^	
ADBB		2m	0.11	−0.03	0.06	0.12	−0.04	0.00	0.04	−0.11	−0.12	0.06	−0.09	−0.15	0.14
		6m	0.15	0.15	0.23^*^	0.22	0.20	0.21^*^	0.12	0.15	0.14	0.13	−0.00	0.11	0.37^***^
		12m	0.04	−0.09	0.10	0.12	0.01	0.16	0.05	0.18	0.27^*^	0.04	0.05	0.18	0.20

* *p < 0.05*.

** *p < 0.01*.

*** *p < 0.001*.

*2m, 2 months, 6m, 6 months, 12m, 12 months*.

## Discussion

Although previous studies have shown that attending medical check-ups with pediatricians trained on the ADBB scale and on a behavioral intervention significantly reduces SSW in full-term infant population (21, 22), no prior research has compared SSW between preterm infants who attended medical check-ups with neonatologists trained in the ADBB scale and in a IGI during their first year of age with those who attended routine pediatric care. Our results suggest that the intervention reduce SSW on the intervention group during their first 12 months of corrected age as observed on the ADBB scores, compared to the control group.

Prior research used the ADBB scale to screen for emotional distress, including preterm population with gestational age at birth between 24 and 36 + 6 weeks, but this is the first clinical trial using IGI by neonatologists themselves. The results of this study concur with previous research, suggesting that moderate and late prematurity is associated with SSW in infancy (8–10, 30).

Regarding parents' mental health, prior research relates depressive symptoms in the mother and both parents' mental health with SSW on infants (2). Our study found positive correlations between SSW at 6 months with mother's depressive and PTSS at 12 months and with father's PTSS at 12 months. This supports bidirectional interplay between SSW and parental psychopathology, at least in the case of mothers, and could indicate an increased risk of parents with preterm infants that evidence SSW to develop PTSS.

The correlation between infants' SSW and parents' mental health could be understood from the perspective of the development of emotional regulation in the infant, as explained by Mäntymaa et al. (2). Considering that emotional regulation is a dyadic (and triadic) process of mutual adaptation, in the case of preterm birth, early stressful life events (such as preterm birth and early hospitalization) suffered by both infant and parents, could overload this adaptation capacity, affecting the variables SSW and parental mental health after the child has been discharged from the NICU.

Alterations in parental mental health can last over 2 years in the case of parents of preterm infants (11). These alterations may restrain the caregivers' ability to adjust their behavioral and emotional states sufficiently to the infant's need for emotional regulation (31). Conversely, preterm infants evidence a higher prevalence of SSW when compared to full-term infants, and “withdrawn” infants, as described by Costa et al. (32), can be less attentive and show less active communication and engagement with their caregivers when compared with non-withdrawn infants. Both the infant and their caregiver must adjust their behaviors and emotional states to meet the needs of each other and the social context (2). In the case of some preterm infants and their parents, this capacity may be affected.

Suppose we understand an infant's SSW as an indicator of the overloaded mutual adaptation capacity of the infant-caregiver dyad (2). In that case, the detection of SSW in clinical contexts should be a sign of alert to examine the parents' mental health. Also, during the first year of the preterm population, and in addition to the screening of postpartum depression symptoms currently being carried out by indication of the Chilean Heal Ministry (33), we could include the assessment of PTSS for parents (both mother and father), considering that they evidence significantly higher levels of PTSS when compared to parents of full-term infants, and that posttraumatic stress disorder (PTSD) has been linked with adverse outcomes in children (11, 12, 34).

Nonetheless, our results emphasize the importance of including a standardized assessment and intervention of early indicators of emotional distress, such as the ADBB scale, on moderate and late preterm follow-up programs as it could reduce the risk for later pathologies (1). This could significantly diminish SSW and increase the capacity to detect child distress key indicators, as shown by Bonifacino et al. (21, 22), and consequently provide new elements to organize mental health as part of an interdisciplinary intervention of follow-up programs.

These findings also provide additional efficacy and support for an intervention that is carried out without using other instances than the ones provided by the already existing health care setting (such as the preterm follow-up programs), using the routine medical check-ups for screening and reducing socioemotional developmental risks, and to enhance the development of interaction and social skills on the preterm population.

In summary, prematurity (severe or not) can put parent-infant relationships at risk as it hampers the ability of each of them to synchronize with the other. Parents require guidance to read the infant's signals and avoid repetitive mistakes in responding to the infants' clues. Due to this, other behavioral early interventions programs have been implemented as RCTs to improve different emotional and behavioral aspects of preterm infants, their caregivers, and the quality interactions between them (35–37).

## Limitations

Some limitations must be considered when interpreting our results. First, as in other randomized controlled trials in clinical contexts, data loss due to participants not attending medical check-ups was a problem in our study. Although we ran different analyses to overcome this limitation, we still think that this must be considered in future studies. It is even possible to think that including ADBB trained professionals in preterm follow-up programs and including specialists in perinatal mental health could help reduce absences to medical check-ups and resistance from other patients and families.

Second, the assessment of some variables in parents was carried out using self-report, without the evaluation of psychiatry professionals, which could lead to misdiagnosis.

Third, the unbalance of the groups with respect to initial diagnosis should be taken into account. This problem is not necessarily absent in clinical studies, probably due to the reliance on randomization or, as in this case, due to the timing of the study. It was not possible to randomize the participants considering their initial diagnosis since the first ADBB assessment was at two months after birth and recruitment was during the hospitalization time (that is, immediately after birth).

The imbalance between groups could have an effect on the outcome variable after treatment. It is interesting to consider that this effect could favor both the intervention and control groups. For example, as Wei and Zhang [(38), p. 1202] state, “Intuitively speaking, the group with better baseline values may have an advantage when comparing posttreatment values since it was already better at baseline”. However, imbalance must be taken into account and these results should be understood as a first step toward the understanding of the effectiveness of guidance as an intervention in premature infants.

## Conclusion

Prior evidence suggests that IGI intervention is efficient in terms of cost-effectiveness because it uses existing resources and scenarios, such as legally established medical check-ups. Along with this, there is no evidence of possible risks in its application, nor did we see them in this study.

Implementing a detecting-intervention device such as the ADBB screening and the IGI intervention, could lead to clinically relevant improvements by decreasing early emotional distress indicators and therefore reducing alterations in the psychological development of this risk population. Considering the described limitations, the results of this clinical trial suggest that detecting SSW and performing an IGI intervention in three medical check-ups during the first year of corrected age decrease SSW behaviors and may enhance emotional development over time.

Evidence suggests that interactive guidance may be able to help parents and infants under challenging situations, as well as improve developmental outcomes in premature babies. SSW is a risk for development, since it communicates that the child cannot cope with certain situations and is therefore in need of help. This study seeks to present a new helping tool when sustained social withdrawal is detected. Considering the lack of specialized help, be it psychological or psychiatric, the implementation of guidance by pediatricians and neonatologists in charge of the follow-up of preterm is essential.

Further investigation will be required to make these results more robust. For example, can interactive guidance intervention lower the risk of future psychopathology on preterm infants? If it can, which pathologies could be prevented and which ones would be resistant?

## Data Availability Statement

The raw data supporting the conclusions of this article will be made available by the authors, without undue reservation.

## Ethics Statement

The studies involving human participants were reviewed and approved by Comite Ético Científico de la Unidad de Investigación y Ensayos Clínicos de Clínica Alemana de Santiago (Chile). Written informed consent to participate in this study was provided by the participants' legal guardian/next of kin.

## Author Contributions

JB conceived and designed the study, drafted the manuscript, and coordinated the research teams of both health centers. MP participated in the study design, provided critical review, and coordinated the Clinica Alemana de Santiago research team. AM-S participated in the study design, planned the statistical analysis and provided critical review. AG and SS participated in the study design and provided critical review. HC, JG, and LM, provided critical review. RS participated in the manuscript and provided critical review. MM coordinated Hospital San Jose research team and provided critical review. All authors read and approved the final manuscript.

## Funding

This study received funding from the Academic and Scientific Department of Clinica Alemana de Santiago. The funder was not involved in the study design, collection, analysis, interpretation of data, the writing of this article or the decision to submit it for publication. Clinica Alemana de Santiago is part of a nonprofit Chilean-German charity corporation and adheres to the Good Clinical Practices and the requirements of the Public Health Institute of Chile in accordance with their current Guide of Inspection of Clinical and Pharmacological Studies.

## Conflict of Interest

The authors declare that the research was conducted in the absence of any commercial or financial relationships that could be construed as a potential conflict of interest.

## Publisher's Note

All claims expressed in this article are solely those of the authors and do not necessarily represent those of their affiliated organizations, or those of the publisher, the editors and the reviewers. Any product that may be evaluated in this article, or claim that may be made by its manufacturer, is not guaranteed or endorsed by the publisher.
